# Facilitating preprint sharing in the health sciences

**DOI:** 10.1371/journal.pmed.1004051

**Published:** 2022-06-21

**Authors:** Marcel LaFlamme, Raffaella Bosurgi

**Affiliations:** Public Library of Science, San Francisco, California, United States of America

## Introduction

Earlier this year, a study of the effects of heterologous boost immunization against COVID-19 was published in *PLOS Medicine* [1]. The study reports that, for individuals previously receiving one dose of the adenovirus-vectored Convidecia vaccine, a booster vaccination with a different protein-subunit-based vaccine is well tolerated and stimulates an enhanced immune response. These findings contribute to a broader base of evidence about how different COVID-19 vaccines can be optimally and safely deployed [2]. Yet this study is also notable because it was the first *PLOS Medicine* article whose authors opted in, at the point of submission, to sharing a preprint of their manuscript through an expanded partnership with the preprint server medRxiv.

A preprint is a version of a scientific manuscript posted on a public server prior to or in parallel with formal peer review. Time to publication in a traditional journal can be lengthy, as submissions make their way through rounds of rigorous review. This means that by the time an article is published, science may well have moved on, especially in rapidly evolving contexts like the COVID-19 pandemic. Sharing preprints can accelerate scientific progress, by enabling others to critically engage with and build on the latest results. For individual researchers, posting a preprint is a way to invite formative feedback and to increase the visibility of one’s work. Preprints also model a culture of trust in scholarly communication, opening the door to new collaborations rather than fixating on concerns about being “scooped.”

But are preprints a good fit for the health sciences? With the launch of arXiv in 1991, it was physics researchers who created a public online repository for unpublished manuscripts rather than circulating them privately via email or, before that, postal mail. In the years that followed, parallel efforts popped up in other fields, including a repository for clinical research created by *BMJ* in 1999 and later shuttered due to low usage [3]. As preprint sharing in the life sciences has increased over the past decade, an extensive debate has unfolded about applying this practice to medical research, given the risks of propagating erroneous information that stands to have an impact on human health [4]. Still, as the benefits of preprint sharing (and the reality that peer review is no panacea for error) are weighed, journal policies on preprints have become more progressive; by 2020, 85 of the 100 top-ranked clinical journals joined *PLOS Medicine* in agreeing to consider submissions that had previously been posted as a preprint [5]. There is also clear evidence that the COVID-19 pandemic led to an uptick in preprint sharing by health science researchers, with as many as 5,000 COVID-19 preprints posted in a single month at its peak [6]. As these numbers stabilize and as attention turns to next steps, the time has come for *PLOS Medicine* to take a more proactive role in facilitating this open science practice.

## PLOS and medRxiv

In 2019, Cold Spring Harbor Laboratory (CSHL) launched medRxiv, a dedicated preprint server for manuscripts in the medical, clinical, and related health sciences. Built as a companion to bioRxiv, CSHL’s widely used platform for life science preprints, medRxiv quickly became the community solution of choice for preprint sharing. PLOS was one of the first publishers to allow authors with a preprint on medRxiv to transfer their manuscript directly to relevant PLOS titles for publication consideration. To date, *PLOS Medicine* has received more than 300 submissions in this way, several of which have been accepted and published in the journal.

More recently, the journal has expanded its partnership with medRxiv to make preprint sharing even easier for *PLOS Medicine* authors. As of January 6, 2022, authors completing a full submission to the journal have been given the option to have PLOS forward their manuscript to medRxiv for posting as a preprint [7]. This offering builds on the success of a similar arrangement with bioRxiv involving six other PLOS titles, which has resulted in more than 9,000 authors opting in to have their life science manuscripts forwarded to bioRxiv at the point of submission. Services like this, which center author choice and focus on lowering barriers to participation, have proven to be effective methods for accelerating the uptake of open science.

In the context of the health sciences, PLOS has particularly emphasized the importance of responsible preprint sharing. Indeed, four years ago the editors of *PLOS Medicine* articulated a set of principles for the early sharing of research with implications for human health [8]. These included transparency in reporting, clarity about the role of a preprint versus a peer-reviewed journal article, and responsibility for safety. Today, we are confident that *PLOS Medicine*’s expanded partnership with medRxiv satisfies each of these principles. The journal’s own standards for reporting align with medRxiv’s requirements for funding sources, competing interests, and ethics approvals to be declared, as well as clinical trials to be registered. Each preprint posted on medRxiv bears a prominent notice alerting readers to the manuscript’s peer review status, and a link to the published article is automatically added once it becomes available. Each preprint is also screened by researchers with related expertise to ensure that posting will not pose a risk to patients or public health.

So far, 18% of *PLOS Medicine* authors completing a full submission to the journal have opted in to having their manuscript considered for posting on medRxiv. This level of adoption is comparable to opt-in rates at other PLOS journals for posting to bioRxiv, and suggests that the facilitated posting service is valued by health science researchers (even as we hope to see even more of our authors take advantage of it). It’s worth noting that the absolute number of *PLOS Medicine* manuscripts getting posted to medRxiv is still small, because at present the journal is offering the facilitated posting service only to those authors whose manuscripts will be sent out for peer review. In the future, we may consider extending the service to all authors at initial submission. While we explore new ways of unlocking the value for science that preprints offer, *PLOS Medicine* remains committed to understanding community needs and to maintaining a balance between rapid dissemination and expert curation that our readers can trust.
